# Relationship between blood amyloid A and resting magnetic resonance functional brain connections in patients with obstructive sleep apnea–hypopnea syndrome

**DOI:** 10.1007/s11325-022-02613-2

**Published:** 2022-05-03

**Authors:** Xiang Wang, Zhiyu Bai, Yaqing He, Qiaozhen Wu

**Affiliations:** Department of Respiratory, Suzhou Ninth Hospital Affiliated to Soochow University, Suzhou Ninth People’s Hospital, Suzhou, 215200 China

**Keywords:** Obstructive sleep apnea–hypopnea syndrome, Serum amyloid A, Magnetic resonance imaging, Rest state function connection, Correlation

## Abstract

**Objective:**

The aim of this study was to analyze the relationship between serum amyloid A (SAA) concentrations in patients with obstructive sleep apnea–hypopnea syndrome (OSAHS) and their magnetic resonance imaging (MRI) of resting brain function.

**Methods:**

Male patients with OSAHS were enrolled from January to June 2019 in Suzhou Ninth People’s Hospital Affiliated to Soochow University, and nineteen healthy male volunteers were selected as the normal control group. The patients with OSAHS were divided into mild, moderate, and severe groups according to their apnea-hypopnea index (AHI). Early in the morning after the polysomnography (PSG), blood samples were collected and serum levels of serum amyloid A (SAA) were measured by enzyme-linked immunosorbent assay. All subjects were scored by the Auditory Verbal Learning Test (AVLT) scale. Resting brain function images of healthy male volunteers and patients in the severe group were collected by 3.0 T magnetic resonance scanner. SPSS25.0 software was used for statistical analysis.

**Results:**

The SAA of the OSAHS group (*n* = 43) were higher than those of control group (*n* = 19). The scores of AVLT-immediate and AVLT-delay in the severe OSAHS group were lower than those in the control group (*P* < 0.05), and it was negatively correlated with SAA. In the severe OSAHS group, the rest state Function Connection (rsFC) in temporal lobe, marginal lobe, and frontal lobe was lower than that in the control group (*P* < 0.05) and was significantly negatively correlated with SAA. The rsFC in bilateral parietal lobes was higher than that in the control group (*P* < 0.05), was significantly positively correlated with SAA, and was negatively correlated with AVLT-delay.

**Conclusions:**

The significant increase in SAA concentration in patients with OSAHS correlated with brain rsFC intensity, providing a reference role for the diagnosis, treatment, and prognosis of cognitive dysfunction in patients with OSAHS.

## Introduction

Obstructive sleep apnea–hypopnea syndrome (OSAHS) is a chronic respiratory disease of sleep characterized by recurrent hypopneas and apneas caused by partial or complete obstruction of the upper airway. OSAHS causes intermittent hypoxemia, repeated micro-arousals, and sleep structure disorders, and is associated with multi-system complications [1]. According to foreign statistics, the prevalence rate of ADULT OSAHS is about 4%, while in China, the prevalence rate of adult OSAHS is about 12–13%, with an increasing trend [2].

Cognitive dysfunction is the main complication of the nervous system in patients with OSAHS [3, 4]. OSAHS significantly increases the concentration of SAA, an inflammatory response factor. Studies have found that SSA is an acute phase inflammatory protein associated with Alzheimer’s disease [5]. One study has found that SAA can be used as a potential peripheral blood marker to predict cognitive impairment in patients with vascular dementia [6]. Relevant studies have shown that the SAA level significantly increases in the serum of patients with OSAHS, that the increase is positively correlated with the severity of OSAHS, and the SAA level significantly decreases after CPAP treatment [7].

Resting-state functional magnetic resonance imaging (rs-fMRI), based on blood oxygenation level dependence (BOLD), is an important technology in studying cognitive impairment with an ability to detect brain structure and function noninvasively. Studies have shown that patients with OSAHS have an abnormal resting-state default network, including changes between internal functional connections [8–10]. However, there is a lack of research on the correlation between SAA and functional connectivity of brain regions in patients with OSAHS.

In this study, we aimed to analyze the relationship between SAA, resting-state brain functional connectivity, and cognitive function. 

## Materials and methods

### Research object

From January to June 2019, male patients with OSAHS were selected from outpatients or inpatients in Suzhou Ninth People’s Hospital affiliated with Soochow University, and were further divided into a mild group, moderate group, and severe group according to AHI. Healthy male volunteers were selected as the normal control group. All selected patients and controls met their respective trial inclusion criteria.

The inclusion criteria of patients with OSAHS were as follows: (1) patients with OSAHS diagnosed by PSG had no other sleep disorders; (2) no history of airway and throat surgery, such as tonsillectomy and adenoidectomy; (3) age ≥ 20 years old and ≤ 60 years old; (4) right-handedness; (5) no alcohol addiction or illegal drug use; (6) no use of psychotropic drugs or medication which may affect on oxygen saturation, such as selective serotonin reuptake inhibitors; (7) no organic brain diseases and no serious cognitive impairment, such as meningioma, pituitary tumor, Parkinson’s disease, and Alzheimer’s disease, and scores on Minimum Mental State Examination (MMSE) > 24/30; (8) no lung disease or other chronic inflammatory diseases by CT examination and pulmonary function test, such as chronic bronchitis; (9) no MRI scan contraindications (no metal stents, no metal dentures, no pacemaker).

The inclusion criteria of the normal control group were as follows: (1) no OSAHS or other sleep disorders confirmed by PSG; (2) age, education background, and handedness were matched with the selected patients with OSAHS; (3) healthy, without any history of neurological diseases, psychiatric medication, or lung disease; (4) no contraindications to MRl examination. This study was reviewed by the Ethics Committee of Suzhou Ninth Hospital Affiliated to Soochow University (No.2019005), and all subjects signed informed consent.

### PSG

Night PSG was carried out in the sleep monitoring Center of Suzhou Ninth People’s Hospital. Subjects were instructed to avoid behaviors including smoking, drinking alcohol, drinking coffee, taking sedative psychotropic drugs that may affect the monitoring process or outcomes within 24 h before the PSG examination. All PSG monitoring was performed with the Alice 6 polysomnography monitoring system of Philips Wellcome, USA. All subjects slept at least 7 h during the PSG; monitoring content included electrocardiogram, electroencephalography, electroophthalmography, electromyography, oral and nasal breathing airflow, chest and abdominal excursions, snoring and blood oxygen saturation. All data were stored, then automatically processed by computer software, and manually corrected by a professional sleep technician. According to the AASM manual definition, OSAHS was diagnosed when more than five respiratory obstruction events per hour were scored. Sleep stages were classified according to the AASM manual with respiratory events scored using standard criteria [11]. Specifically, apneas were scored by an event lasting 10 s or more with flow amplitude decreased by 90% or more. Similarly, hypopneas lasted 10 s or more and had a decreased flow amplitude of 50% or more associated with a decrease in oxygen saturation of 3% or an arousal.

### Serum sample collection and SAA concentration detection

For all subjects, 2 ml fasting venous blood was collected on the morning after PSG monitoring. Blood samples stood at room temperature for 2 h and centrifuged at low speed for 5 min, collected and SAA concentration was determined by ELISA. According to instructions of the ELISA kit, the concentration of SAA in peripheral blood was quantitatively detected by double-antibody sandwich method. The operational steps were carried out in strict accordance with the instructions, and the absorbance value was measured by multifunctional microplate meter (O.D.) to calculate the concentration of SAA in the sample according to the O.D.

### Related scale scores for the subjects

(1) ESS (Epworth sleepiness scale) this questionnaire was used to assess daytime sleepiness of the subjects conducted before PSG examination. A total score of *ESS* ≥ 9 was considered sleepiness. (2) The Mini-Mental State Examination (MMSE) excluded subjects with significant cognitive impairment and senile dementia; (3) Auditory Verbal Learning Test (AVLT) included immediate recall and delayed recall used to evaluate the memory function of subjects. The total score of immediate memory is 75, delayed memory is 15, the bound score is 15, immediate memory and delayed memory a 4, and the lower the score, the worse the memory.

### MRI data acquisition and fMRI data processing in resting state

Data were collected using a 3.0 T magnetic resonance scanner (GE Discovery MR750 3.0 T) with a standard head 24 channel phase control coil. The data were preprocessed by the APABI software on a MATLAB platform. Functional connection analysis: at the resting state, the posterior cingulate cortex (PCC) of the human brain is one of the most intense areas of brain activity at the resting state [12]. Therefore, the cingulate cortex (MNI coordinate (3, − 57,26) [13] was taken as the center, and the radius of 6 mm was taken as the region of interest. Resting-state fMRI Data Analysis Toolkit (http://resting-fMRI.sourceforge.net) was used to extract BOLD sequence in the resting region of interest, and voxelwise was applied to do correlation analysis with other regions of DMN, so as to obtain brain regions with significant connections to region of interest. Two groups of data were obtained after the single sample *T* test, and the two groups of brain regions were selected as the MASK during the double-sample *T* test. When making multiple comparisons, the AlphaSim program provided by REST was used to correct the resulting positive result image (*P* < 0.05) presented by the REST software. During the correlation analysis, the REST software was used to extract the *Z* value of the brain region changed by rsFC, and the correlation analysis was conducted with AHI, ODI, ESS, MMSE, SAA, AVLT-immediate, and AVLT-delay.

### Statistics

SPSS 25.0 statistical software was used for statistical analysis. Data are expressed as means ± standard deviation. Homogeneity of variance testing was performed before comparisons were conducted. Age, years of education, BMI, ESS score, AHI, ODI, MMSE score, AVLT-immediate score, AVLT-delay score, serum SAA concentration level, and rsFC between the two groups were examined by independent sample *t* test. In patients with severe OSAHS, correlation analysis was performed between the rsFC abnormal brain areas and SAA, ESS score, AHI, ODI, MMSE score, AVLT-immediate, and AVLT-delay score. *P* < 0.05 was considered statistically significant.

## Results

### General information

In this study, all patients were male, and there was no statistical difference in age, education, and MMSE between the 43 patients with OSAHS and the 19 normal controls (*P* > 0.05). Compared with the normal control group, BMI, AHI, ODI, and ESS of the OSAHS group were significantly greater (*P* < 0.05). Specific results are shown in Table 1.

**Table 1:** Comparison of general data and PSG parameters between the two groups

Clinical indicators	OSAHS group (43 cases)	Control group (19 cases)	*t*	*P*
Age	39.5 ± 10.4	28.2 ± 10.3	0.448	0.656
Education (years)	11.6 ± 2.8	12.8 ± 2.6	1.534	0.130
BMI (kg/m^2^)	29.6 ± 5.0	22.5 ± 2.7	7.119	0.000
AHI (times/h)	49.6 ± 35.2	2.7 ± 1.4	8.715	0.000
ODI (times/h)	46.3 ± 34.7	1.3 ± 0.6	8.527	0.000
MMSE	28.0 ± 1.3	28.4 ± 1.1	− 1.352	0.181
ESS	11.0 ± 5.6	5.1 ± 2.4	5.856	0.000

*BMI*, body mass index: *AHI*, Apnea–Hypopnea Index: *ODI*, oxygen index: *MMSE*, Mini-Mental State Examination: *ESS*, Epworth sleepiness scale.

### Auditory Verbal Learning Test (AVLT) scale score

Overall comparison of AVLT-immediate and AVLT-delay in each group showed significant differences (*P* < 0.05). Compared with normal control group, there were significant differences between AVLT-immediate and AVLT-delay in the severe OSAHS group (*P* < 0.05). Compared with the mild group, there were significant differences between AVLT-immediate and AVLT-delay in severe OSAHS group (*P* < 0.05). There was no statistical significance between the other two groups (*P* > 0.05). See Table 2 for details.

**Table 2:** Comparison of AVLT-immediate, AVLT-delay, and SAA concentration among the groups

Group	Case	AVLT-immediate	AVLT-delay	SAA μg/ml
Normal control group	19	46.0 ± 6.9	8.1 ± 2.2△	5.67 ± 2.06
Mild OSAHS group	8	46.6 ± 9.6△	7.9 ± 1.5△	12.13 ± 3.52*△
Moderate OSAHS group	10	43.0 ± 5.3	6.4 ± 1.8	18.39 ± 0.76*△
Severe OSAHS group	25	40.5 ± 7.0*	5.7 ± 1.4*	23.91 ± 3.24*

*Was compared with the normal control group, *P* < 0.05; △was compared with severe OSAHS group, *P* < 0.05.

### Serum SAA levels in each group

Overall comparison of SAA concentrations in each group revealed significant differences (*P* < 0.05). Further comparison of LSD between the two groups showed that SAA concentration was significantly increased in the mild OSAHS group (*P* < 0.05) and significantly increased in the moderate and severe OSAHS groups (*P* < 0.01) compared with the normal control group. There were also significant differences between mild and moderate groups, mild and severe groups, and moderate and severe groups (all *P* < 0.01). The concentration of SAA increased with the degree of severity of OSAHS. See Table 2 for details.

### Correlation analysis of SAA

Correlation analysis results showed that SAA concentration in serum of patients with OSAHS was significantly positively correlated with AHI, ODI, and ESS score (*P* < 0.05), and SAA was negatively correlated with AVLT-immediate and AVLT-delay (*P* < 0.05). See Table 3 for details.

**Table 3:** Correlation analysis of SAA concentration level

	Correlation coefficient *r*	*P*	95% confidence interval
AHI	0.822	0.000	0.762 ~ 0.875
ODI	0.810	0.000	0.739 ~ 0.868
ESS	0.715	0.000	0.610 ~ 0.803
AVLT-immediate	− 0.333	0.008	− 0.525 ~ − 0.097
AVLT-delay	− 0.443	0.000	− 0.613 ~ 0.243

### Changes of rsFC in patients with severe OSAHS

**Fig. 1:**
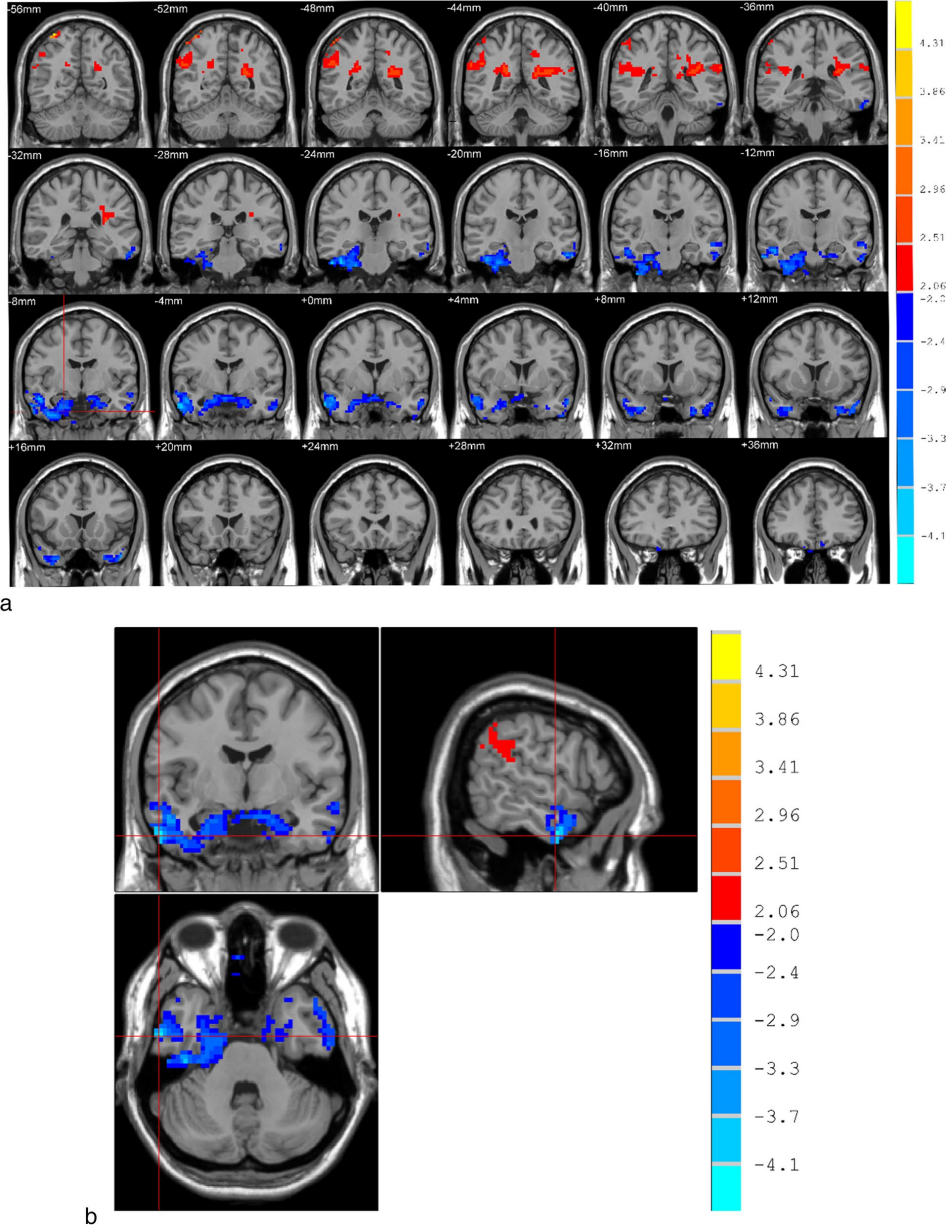

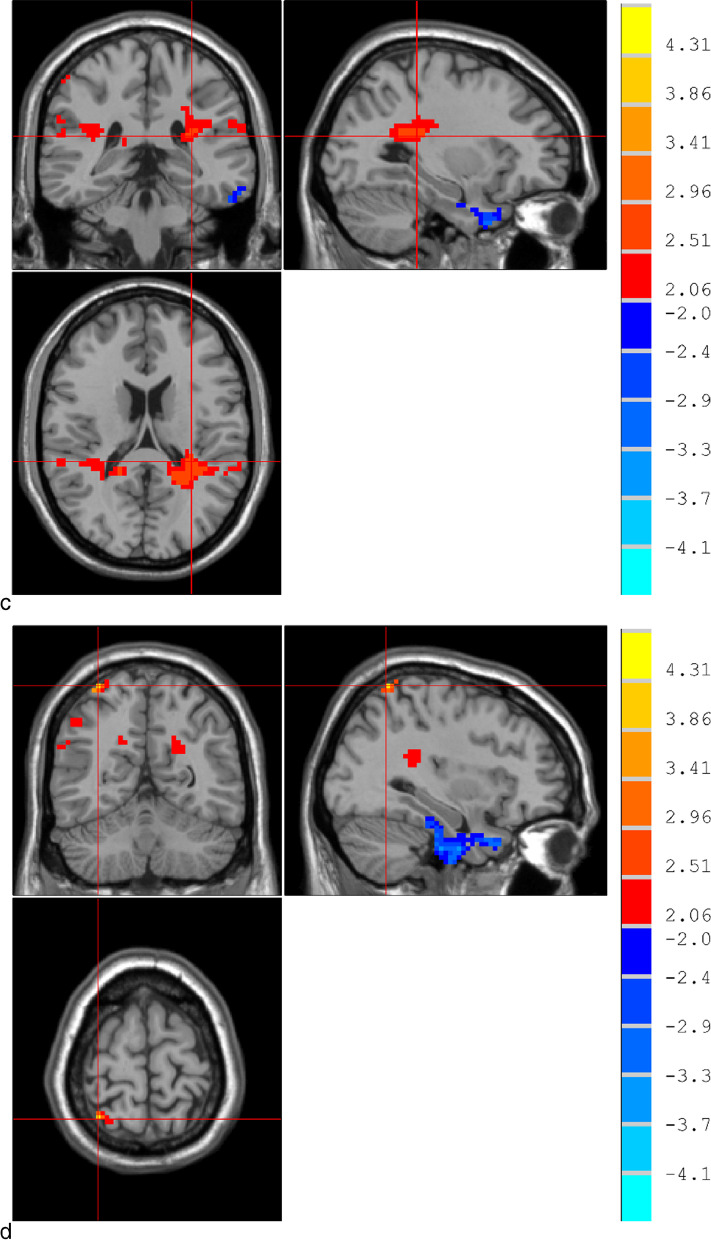
Changes of rsFC in the brain of the severe OSAHS group. Note: Blue-green is the brain region with reduced rsFC, and red-yellow is the brain region with enhanced rsFC.On the left and right of the image are the actual brain on the right and left, respectively, corrected by AlphaSim (*P* < 0.05). **a** The blue areas indicated by the cross cursor are temporal lobe, marginal lobe, and parahippocampal gyrus. **b** The cross cursor points to the blue area of the frontal lobe. **c** The red area indicated by the cross cursor is the left parietal lobe. **d** The yellow and red area indicated by the cross cursor is the right lateral parietal lobe

Compared with the normal control group, the severe OSAHS group showed significant reduction of rsFC in the temporal lobe, marginal lobe, and parahippocampal gyrus (*P* < 0.05); the frontal lobe showed a significant decrease in rsFC (*P* < 0.05), while rsFC in the left parietal lobe and right parietal lobe showed a significant increase (*P* < 0.05). See Fig. 1 and Table 4 for details.

**Table 4:** Brain regions with rsFC changes in patients with severe OSAHS compared with the normal control group

	Brain areas	Voxel	MNI coordinates*	*t*	*P*
rsFC reduces brain areas	Temporal lobe, limbic lobe, parahippocampal gyrus	1403	60 − 6 − 33	− 4.1668	< 0.05
	Frontal lobe	166	− 3 54 − 24	− 3.2733	< 0.05
rsFC enhances brain areas	left parietal lobe	365	− 30 − 36 18	2.9028	< 0.05
	Right parietal lobe	373	33 − 57 69	4.5285	< 0.05

*MNI coordinates, Coordinates of the Montreal Institute of Neuroscience.

### Correlation analysis of rsFC in the brain of patients with severe OSAHS

Bivariate correlation analysis showed that the left parietal lobe was significantly positively correlated with ESS, AHI, ODI, and SAA (*P* < 0.05), while the right parietal lobe was significantly positively correlated with ESS, AHI, ODI, and SAA (*P* < 0.05). The temporal lobe, marginal lobe, and parahippocampal gyrus were significantly negatively correlated with ESS, AHI, ODI, and SAA (*P* < 0.05). The frontal lobe was negatively correlated with ESS, AHI, ODI, and SAA (*P* < 0.05). Temporal lobe, limbic lobe, parahippocampal gyrus, and frontal lobe had no significant correlation with MMSE score, AVLT-immediate, and AVLT-delay. There was no significant correlation between the bilateral parietal lobe in MMSE and AVLT-immediate. The left parietal lobe and the right parietal lobe were negatively correlated with AVLT-delay (*P* < 0.05), respectively. See Table 5 for details.

**Table 5:** Correlation analysis of rsFC in the brain of patients with severe OSAHS

	Left parietal lobe		Right parietal lobe		Temporal lobe, limbic lobe, parahippocampal gyrus		Frontal lobe	
	*r*	*P*	*r*	*P*	*r*	*P*	*r*	*P*
AHI	0.630	0.001	0.647	0.000	− 0.508	0.008	− 0.420	0.033
ODI	0.607	0.001	0.627	0.001	− 0.517	0.007	− 0.427	0.030
ESS	0.419	0.033	0.479	0.013	− 0.572	0.002	− 0.402	0.042
SAA	0.475	0.014	0.524	0.006	− 0.570	0.002	− 0.570	0.002
MMSE	0.114	0.580	0.164	0.425	0.334	0.095	0.138	0.501
AVLT-immediate	− 0.188	0.357	− 0.091	0.660	− 0.043	0.833	− 0.180	0.379
AVLT-delay	− 0.520	0.007	− 0.432	0.028	0.247	0.223	0.320	0.111

### Multivariate linear regression analysis of cognitive dysfunction occurring in patients with severe OSAHS

With cognitive dysfunction as the dependent variable, ODI, ESS, and SAA as independent variables were included in the multiple linear regression analysis model, which showed that the regression model was statistically significant (*F* = 235.633, *P* < 0.001) (Table 6), The independent variable explained 96.6% of the changes in cognitive function, with a high degree of interpretation (Table 7).The results of the significance test showed that the SAA, ODI, and ESS affected the cognitive function in patients with OSAHS (*P* < 0.05) (Table 8).

**Table 6:** ANOVA^a^

Model		Sum of squares	df	Mean square	*F*	Sig.(*P*)
1	Regression	6.304	3	2.101	235.633	0.000^b^
	Residual	0.196	22	0.009		
	Total	6.500	25			

^a^Dependent variable: cognitive dysfunction

^b^Predictors: (constant) SAA, ODI, ESS

**Table 7:** Model summary^b^

Model	*R*	*R* square	Adjusted *R* square	Std. error of the estimate	Durbin-Watson
1	0.985^a^	0.970	0.966	0.094	1.386

^a^Predictors: (constant) SAA, ODI, ESS

^b^Dependent variable: cognitive dysfunction

**Table 8:** Coefficients^a^

Predictors	B	Std. error	Standardized coefficients	Sig.(P)	VIF
(Constant)	4.297	0.045		0.000	
SAA	− 0.032	0.005	− 0.552	0.000	5.057
ODI	− 0.003	0.001	− 0.216	0.015	4.813
ESS	− 0.023	0.006	− 0.267	0.001	3.666

^a^Dependent Variable: Cognitive dysfunction

## Discussion

In this study, seed point correlation analysis found that abnormal changes in resting-state brain functional connectivity occurred in patients with severe OSAHS, with different types of rsFC changes in the temporal, limbic, parahippocampal gyrus, frontal, and parietal lobe. The limbic system areas are related to memory and learning, mainly the cingulate gyrus and hippocampus, and the inferior parietal leaflets in the parietal lobe are related to digital memory and logic. The hippocampus, a core subregion of DMN, is closely related to memory, is very sensitive to hypoxia, and was found to have functional connectivity altered [14] related to cognitive impairment. Research [15] found reduced functional connectivity in the right hippocampus, internal test prefrontal cortex, and left temporal lobe in patients with OSAHS, which is consistent with the results of this study. In this study, there were no statistically different MMSE scores among the subject groups, most likely because the MMSE was not sensitive in detecting mild cognitive dysfunction or early dementia [16].

Although in this study, rsFC in severe OSAHS was significantly reduced, ODI was significantly increased, and AVLT immediate memory and delayed memory were significantly different. No correlation was found between parahippocampus and AVLT immediate memory and delayed memory, so hypoxia appears to lead to hippocampal damage, but this damage has not been transformed into the external performance of cognitive impairment. The subparietal leaflet in the parietal lobe is related to affective memory [17]. This study found that the parietal rsFC was increased and negatively correlated with AVLT delayed memory, so it is reasonable to speculate that parietal rsFC changes triggered cognitive dysfunction and had significant positive correlation with ESS and ODI. Thus drowsiness and hypoxia appear to be two important factors for impairment of the parietal lobe. The frontal lobe is mainly responsible for thinking, execution and is also related to emotion, while the temporal lobe is mainly responsible for processing auditory information and is also related to memory and emotion. The results of this study showed that FC decreased in frontal and temporal lobe in the severe OSAHS group, and ESS score and ODI were significantly higher than the control group. The patients had abnormal functional connectivity due to hypoxia and disordered sleep structure, but there was no obvious correlation with AVLT immediate memory and AVLT delayed memory. This degree of functional change may not cause cognitive and auditory impairment, but it had a significant negative correlation with ESS score, so drowsiness may be an external manifestation of an impaired frontal and temporal lobe.

In this study, the correlation analysis found that SAA was related to cognitive impairment in patients with OSAHS, and associated with abnormalities of functional connectivity in the corresponding brain. Studies have shown that through CPAP treatment, patients with OSAHS improve daytime sleepiness, memory loss, and inattentive concentration, and serum SAA concentration decreased [7] compared with before treatment. However, the positive correlation between SAA and the rsFC speculated that the brain function connection of patients also improved, so SAA may be useful as a biological indicator to evaluate cognitive prognosis in patients with severe OSAHS.

Prior studies [18, 19] have shown that there are gender differences in cognitive function. One study [20] reported that there are cognitive differences between men and women. Therefore, to avoid gender differences, all subjects in this study were male. The study design was cross-sectional, and the sample size was small. No patients with mild or moderate OSAHS were examined by magnetic resonance imaging. These factors are limitations of the current study. 

The findings of this study suggest that intermittent hypoxia of OSAHS may lead to increased levels of SAA and may be associated with impairment of neurocognitive function. Patients with severe OSAHS showed rsFC changes in the temporal, limbic, parahippocampal gyrus, frontal, and bilateral parietal lobes, which may be a neural mechanism related to cognitive impairment in patients with OSAHS. It is possible that the concentration of SAA of patients with OSAHS may indirectly reflect the changes of patients’ cognitive function and brain function. Thus, SAA may have a reference role for the diagnosis and prognosis of cognitive dysfunction in patients with clinically severe OSAHS.

## Data Availability

The datasets generated during and/or analyzed during the current study are not publicly available due to follow-up studies but are available from the corresponding author on reasonable request.
